# Risk profiles and a concise prediction model for lymph node metastasis in patients with lung adenocarcinoma

**DOI:** 10.1186/s13019-023-02288-0

**Published:** 2023-06-20

**Authors:** Shenhua Liang, Yang-Yu Huang, Xuan Liu, Lei-Lei Wu, Yu Hu, Guowei Ma

**Affiliations:** 1 Department of Thoracic Surgery, State Key Laboratory of Oncology in South China, Collaborative Innovation Center for Cancer Medicine, Sun Yat-sen University Cancer Center, Sun Yat-sen University, Guangzhou, P. R. China; 2 Department of Thoracic Surgery, Shanghai Pulmonary Hospital, School of Medicine, Tongji University, Shanghai, P. R. China

**Keywords:** Concise prediction model, Lymph node metastasis, Lung adenocarcinoma, Specimen features

## Abstract

**Background:**

Lung cancer is the second most commonly diagnosed cancer and ranks the first in mortality. Pathological lymph node status(pN) of lung cancer affects the treatment strategy after surgery while systematic lymph node dissection(SLND) is always unsatisfied.

**Methods:**

We reviewed the clinicopathological features of 2,696 patients with LUAD and one single lesion ≤ 5 cm who underwent SLND in addition to lung resection at the Sun Yat-Sen University Cancer Center. The relationship between the pN status and all other clinicopathological features was assessed. All participants were stochastically divided into development and validation cohorts; the former was used to establish a logistic regression model based on selected factors from stepwise backward algorithm to predict pN status. C-statistics, accuracy, sensitivity, and specificity were calculated for both cohorts to test the model performance.

**Results:**

Nerve tract infiltration (NTI), visceral pleural infiltration (PI), lymphovascular infiltration (LVI), right upper lobe (RUL), low differentiated component, tumor size, micropapillary component, lepidic component, and micropapillary predominance were included in the final model. Model performance in the development and validation cohorts was as follows: 0.861 (95% CI: 0.842–0.883) and 0.840 (95% CI: 0.804–0.876) for the C-statistics and 0.803 (95% CI: 0.784–0.821) and 0.785 (95% CI: 0.755–0.814) for accuracy, and 0.754 (95% CI: 0.706–0.798) and 0.686 (95% CI: 0.607–0.757) for sensitivity and 0.814 (95% CI: 0.794–0.833) and 0.811 (95% CI: 0.778–0.841) for specificity, respectively.

**Conclusion:**

Our study showed an easy and credible tool with good performance in predicting pN in patients with LUAD with a single tumor ≤ 5.0 cm without SLND and it is valuable to adjust the treatment strategy.

## Background

Lung cancer is the second most commonly diagnosed cancer, with 2,206,771 new cases and 1,796,144 new deaths worldwide, accounting for 11.4% of all new cancer cases and 18. 0% of all new cancer deaths [1]. More than 40% of lung cancer cases are adenocarcinoma and remain the predominant histological subtype of NSCLC [2]. The overall 5-year survival rate of patients with lung adenocarcinoma is as low as 19.4% [3].

The tumor-node-metastasis (TNM) stage is the chief aspect in the development of treatment strategies for patients with lung adenocarcinoma. Adjuvant therapy is not recommended for stage I patients after radical lung resection, while it is advisable for patients with stage II or higher disease [4–6]. Different pN statuses indicate various TNM stages for the same T1 or T2 stage, which significantly affects the postoperative treatment plan [7]. On some occasions, for example, inadequate operative skill, concern for severe complications and life-threatening risk for further lymph node dissection, and the surgeon’s judgment for low risk of lymph node metastasis, SLND is not completed, so the patient lacks true pN staging.

Therefore, there is an urgent need to develop a practical and highly accurate method to assess pN staging in patients without SLND. Of note, several prediction models related to pN staging or lymph node metastasis have been set up [8–13]. Among the previous related studies, the established prediction models either have unsatisfactory performance with C-statistics < 0.8 in the validation cohort [8, 9, 11, 12] or require predictors from complex or costly tests [10, 13]. In contrast, few studies have focused on high-accuracy prediction of pN based on features of cancer specimens for patients with LUAD after lung cancer resection without complete lymph node dissection. Hence, we analyzed the correlation between lymph node metastasis and clinicopathological characteristics of LUAD and developed a pN prediction model to guide management after LUAD cancer resection alone.

## Methods

### Source of data

This was a retrospective, single-center study where clinicopathological information was extracted from the Sun Yat-Sen University Cancer Center (SYSUCC) database by a well-trained clinician and verified and confirmed for authenticity by another clinician. The Ethics Committee of the center approved the study with the approval number B2020-255-01. The requirement for informed consent was waived due to the study’s retrospective nature and the lack of requirement for identification material. The inclusion criteria were as follows: (1) lung cancer resection, including wedge resection, segmentation resection, lobectomy, bilobectomy, or pneumonectomy, combined with SLND at the Department of Thoracic Surgery of SYSUCC between January 1, 2011, and April 27, 2021. (2) detailed pathology report, including components of the cancer specimen, infiltration condition of the nerve tract, visceral pleura, and lymphovascular infiltration. (3) the pathological subtype of the tumor was lung adenocarcinoma. The exclusion criteria were as follows: (1) multiple primary lung cancers, (2) receiving any types of neoadjuvant therapy, and (3) incomplete clinical or pathological data.

Finally, 2,696 eligible patients were enrolled in the study. Using a random assignment approach, a subgroup of 1918 patients was used as the development cohort. The remaining 718 patients constituted the validation cohort and were outside the model-building process.

### Clinicopathological factors

The following items were retrieved from the electronic medical records: age, sex, smoking history, alcohol drinking history, family cancer history, individual cancer history, infiltration condition of the nerve tract, visceral pleural and lymphovascular, tumor location, differentiation, components of cancer specimen, tumor size, and lymph node station status. The pathological evaluation process complied with the International Association for the Study of Lung Cancer(IASLC)/ American Thoracic Society(ATS)/ European Respiratory Society (ERS) lung adenocarcinoma classification system published in 2011 [14]. For simplicity, differentiation was classified into three categories: (1) only highly differentiated components(HD), (2) medium-differentiated components but without low-differentiated components (MD), and (3) with low-differentiated components (LD). Tumor size was defined as size of the tumor in the formalin-fixedspecimen. The SLND of all cases was performed based on the definition suggested by the European Society of Thoracic Surgeons guidelines, where all mediastinal tissue containing the lymph nodes is removed systematically within anatomical landmarks, while at least three mediastinal nodal stations, one of which must be the subcarinal station, should be eliminated as a minimum requirement^(15)^. Thirty-six cases (3 with right lung cancer and 33 with left lung cancer) underwent bilateral mediastinal lymph node dissection by mediastinoscopy. LVI is defined as the presence of malignant cells within vascular or lymphatic spaces after hematoxylin and eosin staining and is similar to PI and NTI.

### Statistical analysis

Univariate analysis was conducted to test the correlation between clinicopathological factors and pN status using a strictly selected statistical method in the development cohort. For categorical variables, we chose Pearson’s χ^2^ to test whether all expected counts under the null hypothesis were greater than 5, continuity correction χ^2^ if one of the expected counts was no less than 1 but less than 5, and Fisher’s exact test if one of the expected counts was less than 1. As for continuous variables, we chose the t-test if two groups of data had normal distribution and variance homogeneity but Wilcoxon rank-sum and signed-rank tests if they were not. To better interpret the categorical variables in the model, they were transformed into dummy variables. Multivariate analysis using logistic regression was performed with statistically significant factors from univariate analysis to adjust for confounding factors and screen for important factors. Two-tailed values of P < 0.05 were considered to be statistically significant. All statistical analyses were conducted using R software (R version 3. 6. 3 [2020-02-29]).

### Model development and validation

Logistic regression was used to build a prediction model for predicting pN status in the development cohort because it is widely applied in two-category targeted variable prediction. There are two main steps in the model development process. Firstly, the backward stepwise algorithm was applied to factors resulting from the univariate analysis to select the factors incorporated into the final model and eliminate redundant factors. During the selection procedure, the Akaike information criterion, an estimator of prediction error, was used to determine whether the selected factors had a minimum prediction error. The selected factors were then analyzed again by multivariable analysis to ensure that all final factors in the model were significant (p < 0.05). Coefficients, odds ratios (OR), and 95% confidence intervals (CI) were calculated for each variable in the final logistic model. Secondly, a risk score (RS) was generated by the summation of covariates multiplied by their coefficients in the model. The Youden index, defined as “sensitivity + specificity − 1”, was measured to determine the optimal RS cut-off value. A validation cohort was used to test the stability of the established model.

### Model assessment

We assessed the model performance in both cohorts by calculating C-statistics, which are equivalent to the area under the receiver operating characteristic curve (ROC) and indicated comprehensive discrimination of the model, sensitivity as the proportion of participants with positive prediction among those with the true positive condition, specificity as the proportion of participants with negative prediction among those with the true negative condition, accuracy as the proportion of participants with true positive prediction and true negative prediction among all participants, positive predictive value(PPV) as the proportion of participants with true positive prediction among those with positive prediction, and negative predictive value(NPV) as the proportion of participants with true negative prediction among those with negative prediction in both cohorts. The sensitivities and specificities at different cut-off values were used to draw the ROC curve to show the prediction performance of the two cohorts. Decision curve analysis (DCA) was used to evaluate the clinical value of the predictor, which can determine whether utilizing the model to make clinical decisions yields benefits over alternative decision criteria at a certain threshold probability. The clinical impact curve was also employed to evaluate the model, and a nomogram was plotted to visualize the relative weights of predictor variables, facilitating and simplifying the usage of the model.

## Results

**Fig. 1:**
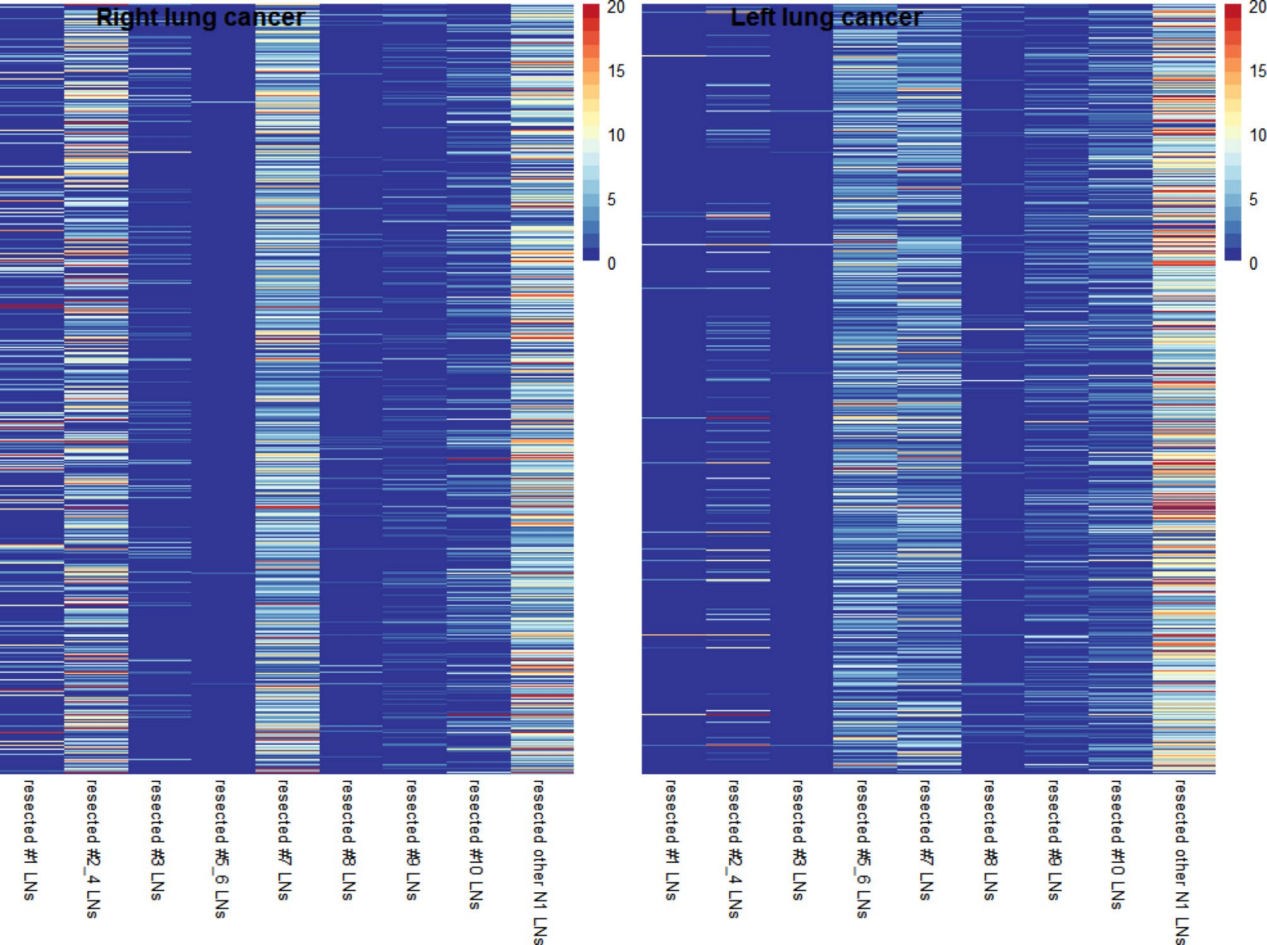
Heatmap for resected nodal numbers in each nodal station for right lung cancer and left lung cancer. One row represents one patient and the column represents the corresponding nodal station

Among participants, 509 patients (18. 88%) accompanied pN1/2 disease(Table 1). In terms of the number of resected lymph nodes, for right lung adenocarcinoma, the highest stations were identified as #2-4 and #7, while for left lung adenocarcinoma, stations #5-6 and #7 had the highest identified numbers (Fig. 1). Table 2 shows that the development and validation cohorts had good homogeneity (p > 0.05 for all clinicopathological factors). The results of the univariate and multivariate analyses of clinicopathological features in the development cohort are listed in Table 3. Eighteen factors, including tumor size, NTI, PI, LVI, RUL, differentiation (MD, LD, HD), 7 kinds of cancer specimen components (acinus, micropapillary, papillary component, solid, lepidic, mucous), four predominant patterns (solid, lepidic, micropapillary, acinus) (all p values < 0.01) correlated with lymph node metastasis in univariate analysis. In the multivariable analysis, eight factors remained significantly correlated: tumor size (p < 0.001), NTI (p = 0.011), PI (p = 0.019), LVI (p < 0.001), RUL (p = 0.001) and micropapillary components (p = 0.024), lepidic (p < 0.001), predominantly micropapillary (P < 0.001). Finally, we obtained nine factors from the backward stepwise algorithm to be incorporated into the ultimate model, as follows: NTI (p = 0.013), PI (p = 0.017), LVI (p < 0.001), RUL (p = 0.001), LD (p < 0.001) and micropapillary components (p = 0.019), and lepidic (p < 0.001), predominantly micropapillary (P < 0.002), and tumor size (p < 0.001) (Table 4).

**Table 1:** Resected nodal numbers and positive patient numbers in each nodal station for right lung cancer and left lung cancer

	Right lung(n = 1604)		Left lung(n = 1092)			
	Resected LN number(mean, range)	Positive patients	Resected LN number(mean, range)		Positive patients	P value
**#1**	1.723(0–29)	32(2.00%)	0.146(0–14)	0(0%)		< 0.001
**#2_4**	5.693(0–44)	120(7.48%)	0.878(0–46)	26(2.38%)		< 0.001
**#3**	0.622(0–13)	25(1.56%)	0.038(0–8)	1(0.09%)		< 0.001
**#5_6**	0.029(0–8)	0(0%)	3.799(0–21)	88(8.06%)		< 0.001
**#7**	5.931(0–40)	105(6.55%)	3.654(0–19)	56(5.13%)		0.149
**#8**	0.243(0–9)	1(0.06%)	0.266(0–10)	5(0.46%)		0.085
**#9**	0.761(0–19)	8(0.50%)	1.302(0–14)	18(1.65%)		0.005
**#10**	1.611(0–21)	58(3.62%)	2.119(0–15)	77(7.05%)		< 0.001
**Other N1**	7.174(0–42)	187(11.66%)	8.658(0–44)	174(15.93%)		0.002
**N1**	8.785(0–56)	203(12.66%)	10.777(0–58)	199(18.22%)		< 0.001
**N2**	15.001(0–92)	194(12.09%)	10.083(0–53)	144(13.19%)		0.435

#1: Right or left low cervical,supraclavicular,and sternal notch lymph nodes; #2_4: right or left upper paratracheal lymph nodes and lower paratracheal lymph nodes; #3:retrotracheal lymph node or prevascular lymph node; #5_6: subaortic lymph node or paraaortic lymph node; #7: subcarinal lymph node; #8: paraesophageal lymph node; #9: pulmonary ligament lymph node; #10: hilar lymph node. Other N1 includes interlobar lymph node, lobar lymph node, segmental lymph node, and subsegmental lymph node.N1 refers to a lymph node metastasis to an ipsilateral hilar or peribronchial lymph node, including #10 and other N1 in this study.N2 refers to a lymph node metastasis to an ipsilateral mediastinal lymph node and/or metastasis to a subcarinal lymph node. P value refers to Chi-square test for difference of positive lymph nodes in each station in right and left lung cancer.

**Table 2:** Clinicopathological features of development and validation cohorts

Features		Development cohort(n1 = 1918)	Validation cohort(n2 = 718)	p value
**Age,mean(range)**		58.15(25–93)	58.55(21–83)	0.3896
**Sex**	Female	1017(53.02%)	415(53.34%)	0.915
	Male	901(46.98%)	363(46.66%)	
**Smoke**	No	1361(70.96%)	573(73.65%)	0.174
	Yes	557(29.04%)	205(26.35%)	
**Alcohol**	No	1623(84.62%)	679(87.28%)	0.088
	Yes	295(15.38%)	99(12.72%)	
**Family ** **cancer history**	No	1496(78%)	598(76.86%)	0.555
	Yes	422(22%)	180(23.14%)	
**Individual cancer history**	No	1833(95.57%)	732(94.09%)	0.128
	Yes	85(4.43%)	46(5.91%)	
**Tumor size**		2.11(0.4-5)	2.15(0.3-5)	0.3970
**NTI**	No	1872(97.6%)	750(96.4%)	0.110
	Yes	46(2.4%)	28(3.6%)	
**PI**	No	1328(69.24%)	542(69.67%)	0.864
	Yes	590(30.76%)	236(30.33%)	
**LVI**	No	1675(87.33%)	672(86.38%)	0.544
	Yes	243(12.67%)	106(13.62%)	
**RUL**	No	1270(66.21%)	516(66.32%)	0.993
	Yes	648(33.79%)	262(33.68%)	
**RML**	No	1783(92.96%)	717(92.16%)	0.519
	Yes	135(7.04%)	61(7.84%)	
**RLL**	No	1558(81.23%)	640(82.26%)	0.568
	Yes	360(18.77%)	138(17.74%)	
**LUL**	No	1431(74.61%)	568(73.01%)	0.417
	Yes	487(25.39%)	210(26.99%)	
**LLL**	No	1630(84.98%)	671(86.25%)	0.436
	Yes	288(15.02%)	107(13.75%)	
**MD**	No	593(30.92%)	257(33.03%)	0.305
	Yes	1325(69.08%)	521(66.97%)	
**LD**	No	1424(74.24%)	566(72.75%)	0.453
	Yes	494(25.76%)	212(27.25%)	
**HD**	No	1819(94.84%)	733(94.22%)	0.578
	Yes	99(5.16%)	45(5.78%)	
**Acinus component**	No	377(19.66%)	147(18.89%)	0.6900
	Yes	1541(80.34%)	631(81.11%)	
**Mp component**	No	1591(82.95%)	646(83.03%)	1.0000
	Yes	327(17.05%)	132(16.97%)	
**Papillary component**	No	1319(68.77%)	537(69.02%)	0.934
	Yes	599(31.23%)	241(30.98%)	
**Solid component**	No	1664(86.76%)	665(85.48%)	0.414
	Yes	254(13.24%)	113(14.52%)	
**Lepidic component**	No	1543(80.45%)	621(79.82%)	0.751
	Yes	375(19.55%)	157(20.18%)	
**Mucous component**	No	1840(95.93%)	741(95.24%)	0.486
	Yes	78(4.07%)	37(4.76%)	
**Papillary predominant**	No	1618(84.36%)	667(85.73%)	0.401
	Yes	300(15.64%)	111(14.27%)	
**Solid predominant**	No	1792(93.43%)	723(92.93%)	0.700
	Yes	126(6.57%)	55(7.07%)	
**Lepidic predominant**	No	1747(91.08%)	698(89.72%)	0.301
	Yes	171(8.92%)	80(10.28%)	
**MP predominant**	No	1878(97.91%)	762(97.94%)	1.0000
	Yes	40(2.09%)	16(2.06%)	
**Mucous predominant**	No	1906(99.37%)	767(98.59%)	0.074
	Yes	12(0.63%)	11(1.41%)	
**Acinus predominant**	No	649(33.84%)	273(35.09%)	0.564
	Yes	1269(66.16%)	505(64.91%)	

**Table 3:** Univariate and multivariate analysis of clinicopathological features in the development cohort

		the development cohort		univariate analysis	multivariate analysis
		pN0(n1 = 1568)	pN1/2(n2 = 350)	p value	p value
**Age,mean(range)**		58.15(25–88)	58.14(25–93)	0.938	
**Sex**	Female	842(53.7%)	175(50%)	0.232	
	Male	726(46.3%)	175(50%)		
**Smoke**	No	1118(71.3%)	243(69.43%)	0.527	
	Yes	450(28.7%)	107(30.57%)		
**Alcohol**	No	1335(85.14%)	288(82.29%)	0.2090	
	Yes	233(14.86%)	62(17.71%)		
**Family cancer history**	No	1216(77.55%)	280(80%)	0.353	
	Yes	352(22.45%)	70(20%)		
**Cancer history**	No	1500(95.66%)	333(95.14%)	0.776	
	Yes	68(4.34%)	17(4.86%)		
**Tumor size**		1.98(0.4-5)	2.72(0.5-5)	< 0.001*	< 0.001*
**NTI**	No	1545(98.53%)	327(93.43%)	< 0.001*	0.011*
	Yes	23(1.47%)	23(6.57%)		
**PI**	No	1136(72.45%)	192(54.86%)	< 0.001*	0.019*
	Yes	432(27.55%)	158(45.14%)		
**LVI**	No	1475(94.07%)	200(57.14%)	< 0.001*	< 0.001*
	Yes	93(5.93%)	150(42.86%)		
**RUL**	No	1013(64.6%)	257(73.43%)	0.002*	0.001*
	Yes	555(35.4%)	93(26.57%)		
**RML**	No	1465(93.43%)	318(90.86%)	0.113	
	Yes	103(6.57%)	32(9.14%)		
**RLL**	No	1278(81.51%)	280(80%)	0.564	
	Yes	290(18.49%)	70(20%)		
**LUL**	No	1181(75.32%)	250(71.43%)	0.149	
	Yes	387(24.68%)	100(28.57%)		
**LLL**	No	1335(85.14%)	295(84.29%)	0.748	
	Yes	233(14.86%)	55(15.71%)		
**MD**	No	388(24.74%)	205(58.57%)	< 0.001*	0.371
	Yes	1180(75.26%)	145(41.43%)		
**LD**	No	1278(81.51%)	146(41.71%)	< 0.001*	0.087
	Yes	290(18.49%)	204(58.29%)		
**HD**	No	1470(93.75%)	349(99.71%)	< 0.001*	NA
	Yes	98(6.25%)	1(0.29%)		
**Acinus component**	No	283(18.05%)	94(26.86%)	< 0.001*	0.400
	Yes	1285(81.95%)	256(73.14%)		
**MP component**	No	1360(86.73%)	231(66%)	< 0.001*	0.024*
	Yes	208(13.27%)	119(34%)		
**Papillary component**	No	1103(70.34%)	216(61.71%)	0.002*	0.261
	Yes	465(29.66%)	134(38.29%)		
**Solid component**	No	1430(91.2%)	234(66.86%)	< 0.001*	0.460
	Yes	138(8.8%)	116(33.14%)		
**Lepidic component**	No	1205(76.85%)	338(96.57%)	< 0.001*	< 0.001*
	Yes	363(23.15%)	12(3.43%)		
**Mucous component**	No	1515(96.62%)	325(92.86%)	0.002*	0.181
	Yes	53(3.38%)	25(7.14%)		
**Papillary predominant**	No	1326(84.57%)	292(83.43%)	0.654	
	Yes	242(15.43%)	58(16.57%)		
**Solid predominant**	No	1504(95.92%)	288(82.29%)	< 0.001*	0.246
	Yes	64(4.08%)	62(17.71%)		
**Lepidic predominant**	No	1399(89.22%)	348(99.43%)	< 0.001*	0.688
	Yes	169(10.78%)	2(0.57%)		
**MP predominant**	No	1556(99.23%)	322(92%)	< 0.001*	0.001*
	Yes	12(0.77%)	28(8%)		
**Mucous predominant**	No	1561(99.55%)	345(98.57%)	0.083	
	Yes	7(0.45%)	5(1.43%)		
**Acinus predominant**	No	494(31.51%)	155(44.29%)	< 0.001*	0.271

NA as a coefficient in a regression indicates that the variable in question is linearly related to the other variables

**Table 4:** Logistic regression coefficients and odds ratio of variables resulted from the stepwise backward algorithm

	Estimate	Std. Error	z value	Pr(>|z|)	OR	2.5% OR	97.5% OR
**(Intercept)**	-3.265	0.205	-15.935	<0.001	0.038	0.025	0.057
**NTI**	0.861	0.348	2.474	0.013	2.366	1.186	4.670
**PI**	0.354	0.148	2.398	0.016	1.425	1.065	1.903
**LVI**	1.906	0.169	11.257	<0.001	6.728	4.837	9.400
**RUL**	-0.528	0.157	-3.351	0.001	0.590	0.431	0.800
**LD**	1.040	0.148	7.035	<0.001	2.828	2.117	3.779
**MP component**	0.405	0.172	2.354	0.019	1.500	1.067	2.096
**lepidic component**	-1.590	0.326	-4.881	<0.001	0.204	0.102	0.371
**MP predominant**	1.354	0.446	3.034	0.002	3.873	1.641	9.506
**tumor size**	0.442	0.072	6.177	<0.001	1.555	1.352	1.790

Std. Error: standard error; OR: odds ratio

**Fig. 2:**
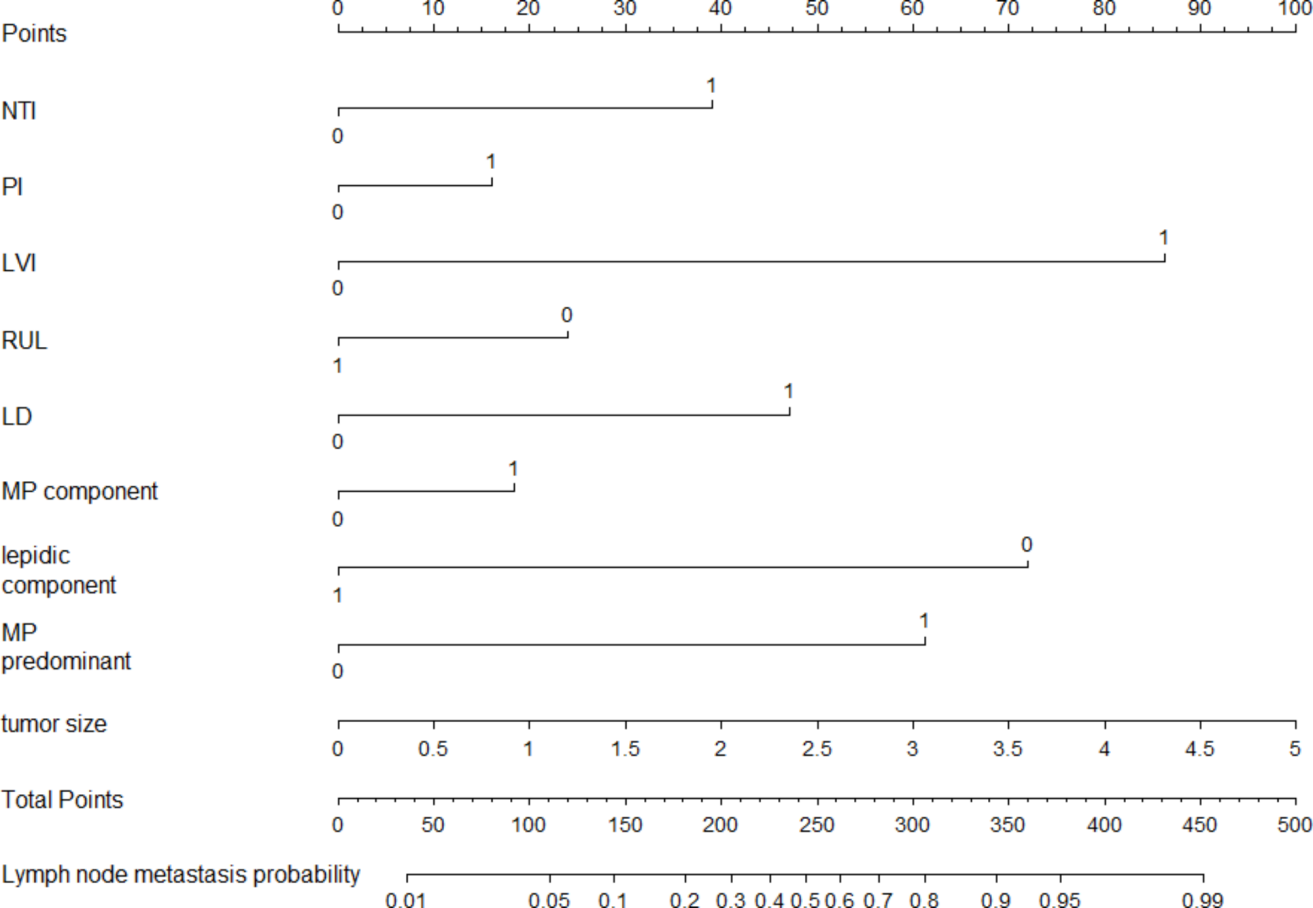
A nomogram to predict the probability of lymph node metastasis of LUAD patients with a single tumor size of < = 0.5 cm. The mark “1” of NTI, PI, LVI,RUL, LD, MP component, lepidic component, MP predominant stands for the present status of the corresponding situation and the “0” for the absent status

**Fig. 3:**
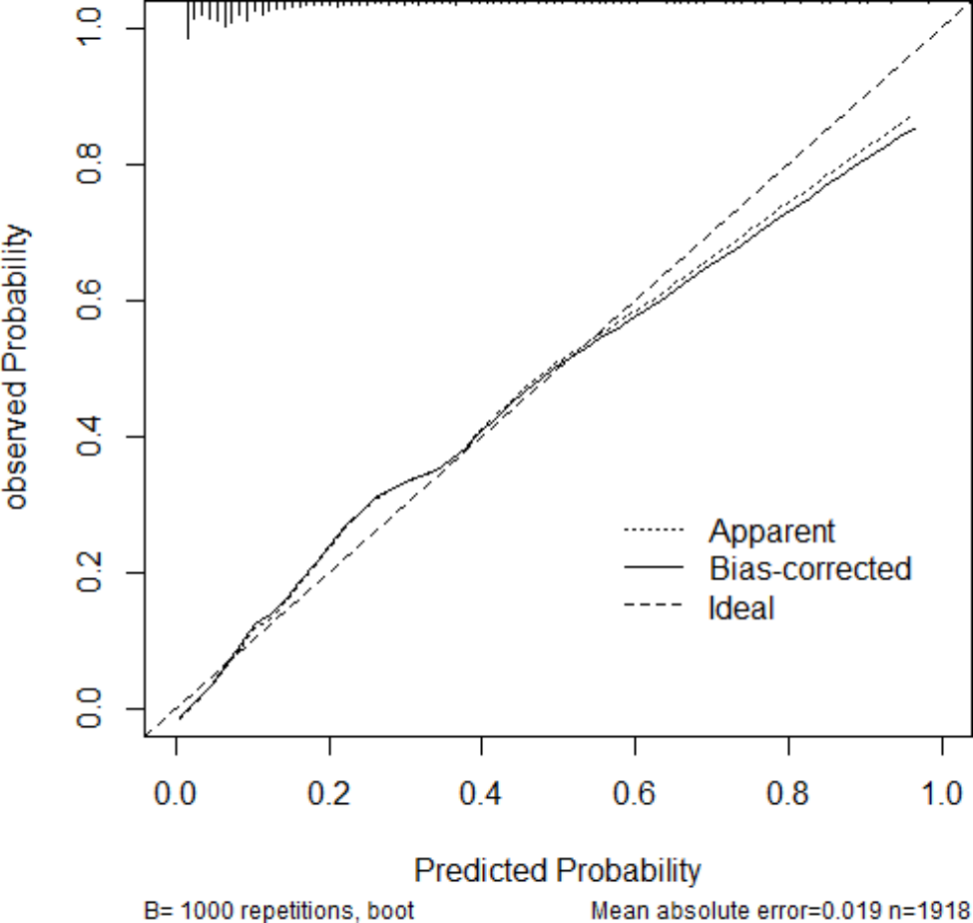
The calibration curve of the prediction model. The X axis is the predicted probabilities measured by the final logistic regression model and the Y axis is the actual probabilities. The calibration curve cohort shows good agreement between the observed outcomes and those predicted

**Fig. 4:**
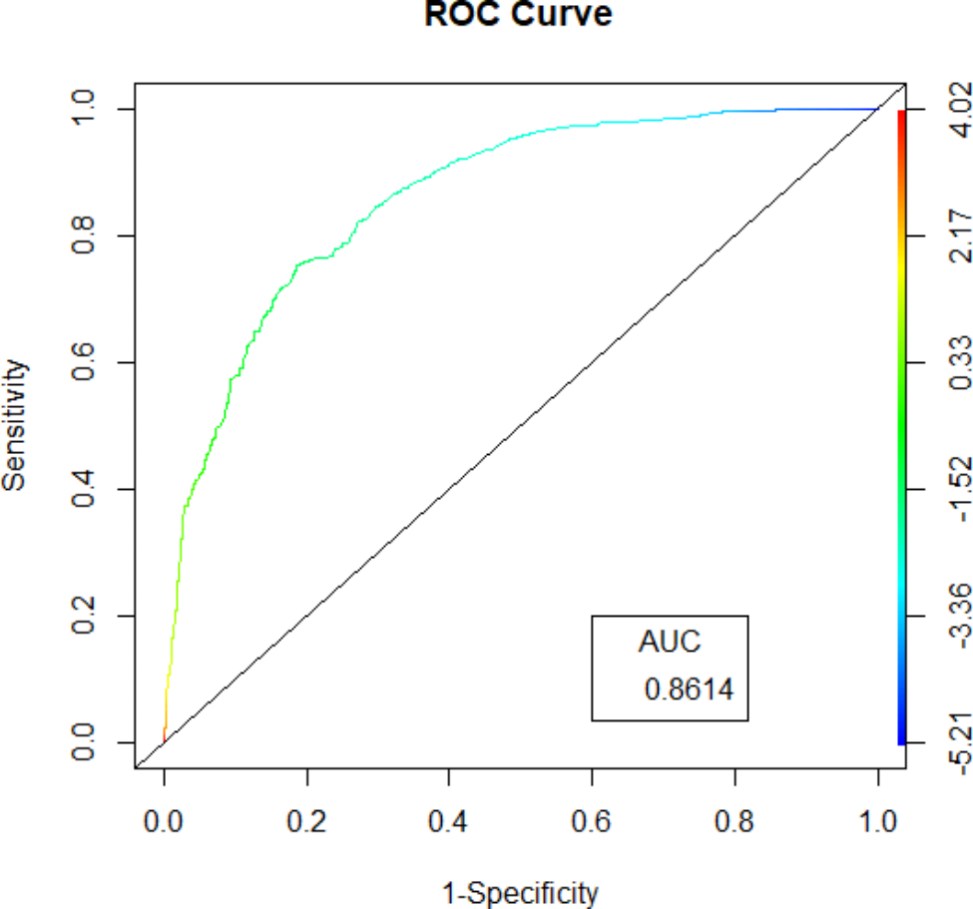
Receiver Operating Characteristics Curve of the prediction model. The area under the receiver operating characteristic curve is greatly significantly better than the noninformative ROC(the diagonal solid line)

**Fig. 5:**
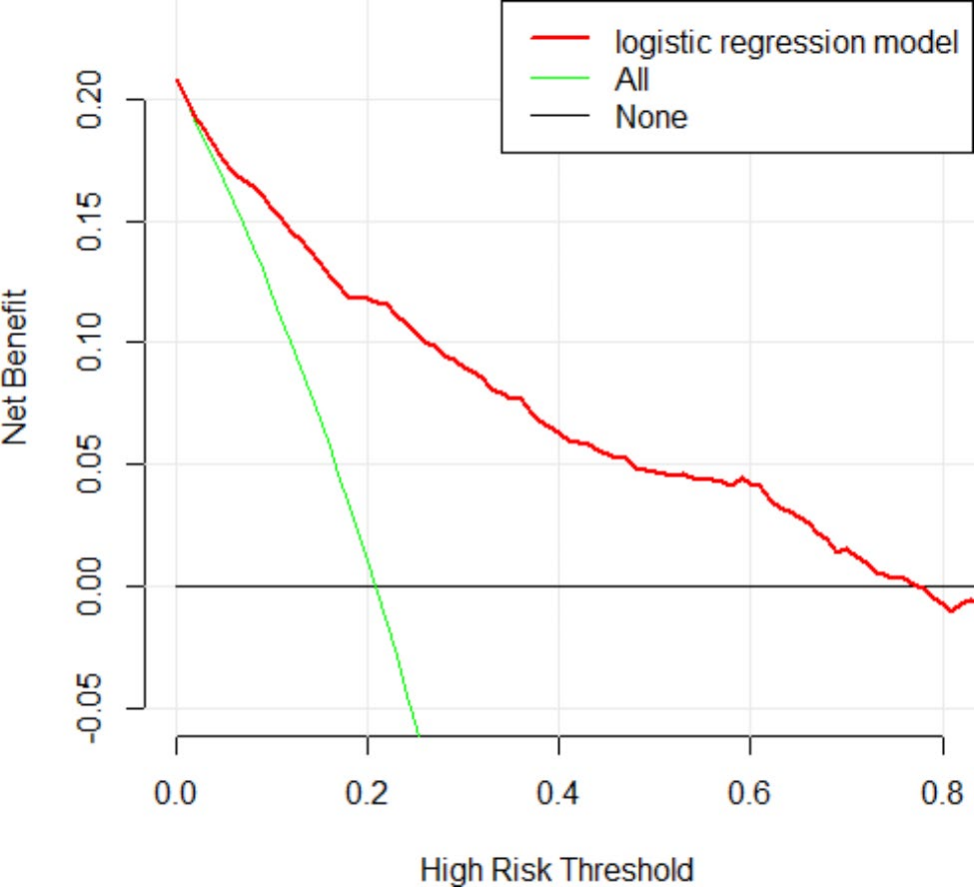
Decision curve analysis of the model. The black horizontal black line is the net benefit of referring none of the patients for lymph node metastasis. The green curve is the net benefit of referring all patients for lymph node metastasis. The red curve represents the logistic regression model

**Fig. 6:**
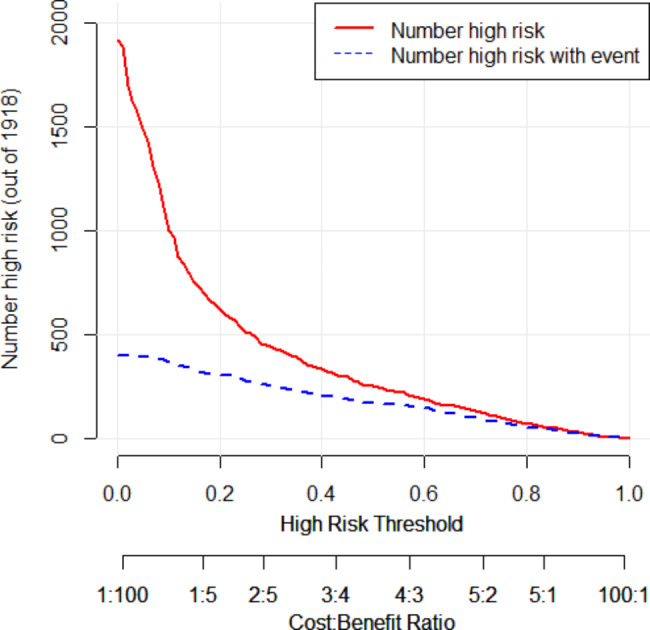
Clinical impact analysis of the model by clinical impact curve. The red solid line represents patients classified as positive (at high risk) by the model while the blue dash line represents patients with true positives at different threshold probability

A novel risk-scoring method for lymph node metastasis prediction was established using the following equation:

$$\begin{array}{c}RS = - 3.265 + 0.861 \times NTI + 0.354\\\times PI + 1.906 \times LVI - 0.527\\\times RUL + 1.040 \times LD + 0.405\\\times micropapillary\,component - 1.590\\\times lepidic\,component + 1.354\\\times micropapillary\,predo\min ant + 0.442\\\times tumor\,size\end{array}$$

where NTI, PI, LVI, RUL, LD, micropapillary component, lepidic component, and micropapillary predominant are two-level categorical variables whose values were 1 for present or 0 for absent, and tumor size is the mean of 3-dimensional lengths of the tumor in centimeters. The likelihood of lymph node metastasis increased with the RS value. By assessing the OR of each factor in the ultimate model, NTI, PI, LVI, LD, micropapillary component, micropapillary predominance, and larger tumor size were favorable for lymph node metastasis, among which LVI was the largest contributor (OR: 6.728, 95%CI: 4.837–9.400), followed by micropapillary predominance, and LD. Location of the right upper lobe (OR: 0.590, 95%CI: 0.431–0.800) and lepidic components (OR: 0.204, 95%CI: 0.102–0.371) decreased the probability of lymph node metastasis in the development cohort analysis. We determined the optimal cut–off RS for the highest Youden index (0.568) as − 1. 430; the C–statistics by the bootstrap resampling method were 0.861 (95% CI: 0.842–0.883), and the sensitivity and specificity were 0.754 (95%CI: 0.706–0.798) and 0.814 (95%CI: 0.794–0.833). In the validation cohort, the C–statistics by the bootstrap resampling method were 0.861 (95%CI: 0.8406–0.8740), and the sensitivity and specificity were 0.686 (95%CI: 0.607–0.757) and 0.811 (95%CI: 0.778–0.841) (Table 5). A nomogram based on the logistic regression model shown in Fig. 2 is convenient for model interpretation and usage. In the nomogram, the value of a certain indicator produces a corresponding point by drawing a vertical line to the point axis from the location of the indicator value. The probability of lymph node metastasis was calculated according to the total number of points. The calibration curve (Fig. 3) of the development cohort showed good agreement between the observed and predicted outcomes. The area under the receiver operating characteristic curve (ROC; Fig. 4) is 0.861, which is significantly better than the non-informative ROC curve (the diagonal solid line). A decision curve is depicted in Fig. 5, considering both the different clinical decision strategies and the prediction model and calculating the net benefit at all possible thresholds. We drew a clinical impact curve to compare the difference between the number of people classified as positive (high risk) and the number of true positives for each threshold probability and measured the cost-benefit ratio at different probability thresholds. At a cut-off value of 0.5 for the probability, the cost-benefit ratio approximately equals 1:1, which can be a reference to other probability thresholds (Fig. 6).

**Table 5:** Model performance in development and validation cohorts

	Development cohort	Validation cohort
**C-statistics**	0.861 (0.842, 0.883)	0.840(0.804, 0.876)
**Sensitivity**	0.754 (0.706, 0.798)	0.686(0.607, 0.757)
**Specificity**	0.814 (0.794, 0.833)	0.811 (0.778, 0.841)
**Accuracy**	0.803 (0.784, 0.821)	0.785 (0.755, 0.814)
**PPV**	0.475 (0.433, 0.517)	0.482 (0.416, 0.550)
**NPV**	0.937 (0.923, 0.949)	0.909 (0.882, 0.932)

PPV : positive predictive value; NPV: negative predictive value

## Discussion

An accurate assessment of the lymph node status involves SLND. Lymph node status in NSCLC, especially pathological status, is important for prognosis and for guiding postoperative therapeutic strategies [15].

Generally, complete lymph node dissection is often indicated and essential for lung cancer resection in most hospitals, especially during surgery for NSCLC with cT1a-2bN0-1M0 [16]. However, this standard of care is not always carried out by thoracic surgeons. Stewart AK et al. [17]found in a survey on lung cancer patient care in 729 hospitals that, among patients receiving surgery, only 57 (8%) of the patients had lymph nodes either sampled or removed from the mediastinum, and as many as 42 (2%) had no mediastinal lymph nodes evaluated. We inferred that the poor performance of patients (advanced age, severe comorbidities, etc.), unskilled operation by the surgeon, and complex anatomy contributed to incomplete resection. Moreover, considering the potential grave complications (such as recurrent nerve palsy, bleeding, chylothorax, and phrenic nerve palsy) and longer operative time, many researchers have focused on whether some patients with lung cancer can forego SLND. Joe B. Putnam, Jr, and his colleagues [18] published results of the ACOSOG Z0030 trial, the largest randomized controlled trial comparing outcome difference between complete lymphadenectomy and further mediastinal lymph node sampling in patients whose systematic and thorough presentation sampling of the mediastinal and hilar nodes is negative. This result showed that mediastinal lymph node dissection (MLND) did not benefit patients with early-stage NSCLC, which indicates that patients with N0 or N1 (less than hilar) confirmed by mediastinoscopy or systematic lymph node sampling during pulmonary resection can help avoid systematic lymph dissection. Conversely, patients without systematic lymph node dissection are at risk of undetected N2 disease. Our study provides a tool to assess N status and predict the probability of lymph node status (N1 or N2 disease).

In this study, we found that NTI, PI, LVI, LD, micropapillary component, micropapillary predominance, and larger tumor size were positively correlated with lymph node metastasis, while the location of the right upper lobe and lepidic component negatively impacted the rate of lymph node metastasis.

It is no doubt that the likelihood of cancer invasiveness and metastasis, including lymph node metastasis, increases as tumor size grows [19–21]. Few studies have focused on the correlation between lymph node metastasis in patients with lung cancer and NTI. However, our study found a positive correlation and first introduced NTI into a prediction model for N status. There are three main methods for carcinoma cell migration: lymphatic vessels, blood vessels, and serosal surfaces, whereas NTI is another ignored approach for carcinoma cell dissemination [22]. The nerve tract possesses a low-resistance plane in the neural sheaths, which serves as a conduit for their migration [23], which may explain the positive correlation between NTI and lymph node metastasis.

PI was considered another factor correlated with lymph node metastasis. This is consistent with studies by Yu et al. [24] and Kudos et al. [25]. Moreover, LVI, LD, micropapillary component and micropapillary predominance, and large tumor size have been well investigated to positively impact lymph node metastasis by many academics [26–28]. RUL is a protective factor against lymph node metastasis. Yi Tan’s study [29] showed similar results that compared to RUL, tumors located in the LLL, LUL, RML, and RLL (ordered by decreasing OR) displayed a higher risk of LNM. Ting [30] reported that in patients with lung adenocarcinoma ≤ 3 cm, the lepidic component was significantly associated with histologic subtype, TNM stage, and lymph node metastasis (P < 0.05), whereas in those with a tumor of greater than 3 cm, this association did not exist.

Several models for predicting lymph node metastasis of lung adenocarcinoma have been previously reported. Zheng [31] developed a radiomics model (RM) using a support vector machine and extremely randomized trees based on 18 F-FDG PET/CT features to predict mediastinal lymph node metastasis. The AUC of RM was 0.81 (95%CI: 0.771–0.848), sensitivity of 0.794, and specificity of 0.704. Keiju Aokage et al. [32] proposed a multivariable logistic regression model based on clinical and radiological factors, leading to C-statistics of 0.8041 and 0.7972, sensitivity of 95.7% and 95.4%, a specificity of 46.0%, and 40.5% for the development and external validation sets, respectively. Zang [33] reported a four-predictor(larger consolidation size, central tumor location, abnormal status of tumor marker, and clinical N1/N2 stage) model for the preoperative prediction of lymph node involvement in patients with clinical stage T1aN0-2M0 non-small cell lung cancer, achieving an AUC of 0.842 and 0.810 in the training and test groups, respectively. The aforementioned models focused on preoperative clinical information and showed moderate discrimination (ACU: 0.7972–0.842), ignoring the critical pathological information from resected specimens considered to be more relevant to lymph node metastasis which was confirmed in our study. Our model merged these key factors and achieved an AUC value of 0.861 (95%CI: 0.842–0.883) and 0.861 (95%CI: 0.8406–0.8740) for the development and validation cohorts, respectively.

These variables can be easily and conveniently obtained from medical records and pathology reports, which involve no additional burden for extra examination or financial cost from patients after surgery. The nomogram is user-friendly because most variables are binomial, except for tumor size, meaning that the user can quickly locate the point of each variable and the possibility of lymph node metastasis according to the sum point. This model is appropriate for patients with resected lung adenocarcinoma specimens of one tumor < 5 cm, including wedge resection. We recommend further adjuvant therapy and more intensive follow-up for the predicted high-risk patients from our model.

Our study had some limitations that should be mentioned. First, it was a retrospective study inherently prone to selection bias, as in all other retrospective studies. Furthermore, its single-center nature lacks generalization, which requires further validation at the multicenter level in the future, although it possesses a large sample and relatively high AUC value. The third limitation was that our model largely depended on the pathological result that could be variable owing to an inter-observer difference. Thus, a pathomics method that automatically extracts and calculates features from a pathological section image may be a potential tool for decreasing inter-observer instability. Most importantly, the true effects of this prediction tool on the treatment strategy and outcome remain opaque, demanding a multicenter, prospective, randomized, controlled study.

## Conclusion

We discovered that NTI, PI, LVI, LD, micropapillary component, micropapillary predominance, and larger tumor size were independent positive predictors of lymph node metastasis in patients with LUAD with a single tumor size ≤ 5 cm after lung resection, whereas RUL and lepidic component were independent negative predictors. The established model could serve as a prediction tool to distinguish patients with pN1/2 from those with pN0 and support treatment strategies for patients with LUAD without SLND.

## Data Availability

The datasets used and/or analysed during the current study are available from the corresponding author on reasonable request.

## List of Abbreviations

CI: Confidence intervals
LD: With low-differentiated components
MD: With medium-differentiated components but without low-differentiated components
HD: With only highly differentiated components
RUL: Right upper lobe
RML: Right middle lobe
RLL: Right lower lobe
LUL: Left upper lobe
LLL: Left lower lobe
MP: Micropapillary
LUAD: Lung adenocarcinoma
LVI: Lymphovascular infiltration
NTI: Nerve tract infiltration
PI: Visceral pleural infiltration
OR: Odds ratios
pN: Pathological lymph node
ROC: Receiver operating characteristic curve
SLND: Systematic lymph node dissection
SYSUCC: Sun Yat-Sen University Cancer Center
TNM: Tumor-node-metastasis
